# Function of the auditory cortex characterized by its intrinsic dynamic coactivation patterns estimated in individuals

**DOI:** 10.1162/IMAG.a.1179

**Published:** 2026-03-24

**Authors:** Maria Hakonen, Kaisu Lankinen, Parker Kotlarz, Jonathan R. Polimeni, Tori Turpin, Jianxun Ren, Danhong Wang, Hesheng Liu, Jyrki Ahveninen

**Affiliations:** Athinoula A. Martinos Center for Biomedical Imaging, Department of Radiology, Massachusetts General Hospital Charlestown, Boston, MA, United States; Department of Radiology, Harvard Medical School, Boston, MA, United States; Harvard-MIT Program in Health Sciences and Technology, Massachusetts Institute of Technology, Cambridge, MA, United States; Division of Brain Sciences, Changping Laboratory, Beijing, China; Biomedical Pioneering Innovation Center (BIOPIC), Peking University, Beijing, China

**Keywords:** auditory cortex, 7T fMRI, coactivation patterns, dynamic functional connectivity, individual-level, human

## Abstract

Determining the functional organization of the auditory cortex (AC) has been difficult with conventional task-based approaches due to the broad responsiveness of auditory subregions to various acoustic properties. Moreover, most studies have investigated functional organization of AC with static methods, although the brain has shown to be organized into dynamic networks. Here, we investigated dynamically varying coactivation patterns of the local networks in the auditory cortex (AC) determined from 7T fMRI data. Dynamic AC patterns successfully captured interindividual variability, as indicated by significantly higher variability between than within individuals for the AC pattern occurrence rates and spatial topographies. The coactivation patterns shared similarities between resting-state and auditory-task data, as indicated by the group-level similarity of 0.84 and individual-level similarity of 0.71 in the spatial topographies. Furthermore, the occurrence rates of AC patterns identified in the task data, using pattern templates derived from resting-state data, correlated with specific task contrast regressors. Our results suggest that the AC function can be characterized by a set of dynamically varying coactivation patterns. These patterns are consistently observed during resting state and auditory stimulation, and they become synchronized with auditory inputs. These findings enhance our understanding of the relationship between spontaneous and stimulus-driven activity in the AC and support the development of more time-efficient paradigms for studying its functional organization.

## 1 Introduction

In comparison to the detailed mapping of other sensory domains, the exact functional organization of the human auditory cortex (AC) has been difficult to characterize. Neurons at different parts of AC are driven by a great variety of stimulus attributes (Bizley et al., 2009; Carruthers et al., 2013; Chambers et al., 2014). Exhaustive testing of all potential feature combinations to examine the functional properties of different AC areas is, thus, difficult even in animal models, not to mention human fMRI studies. A typical human neuroimaging study, therefore, manipulates only a few stimulus dimensions (e.g., frequency, source location, semantic content) for testing a limited set of hypotheses. As a result, the influence of other, untested stimulus dimensions—which might, in fact, drive responses in a given AC area even more strongly—cannot be fully ruled out. A small number of fMRI studies have approached this problem by modeling responses to large collections of natural sounds (Moerel et al., 2013, 2017; Norman-Haignere et al., 2015). For example, a 3T fMRI study (Norman-Haignere et al., 2015) utilized multivoxel decomposition methods to find a set of canonical components from responses to 165 different natural sounds, corresponding to six different, but partially overlapping, functional subsystems of AC. Analogous methods, which infer the response dimensions from the structure in the data, were also utilized to tease apart AC areas with simple vs. complex spectral preferences from 7T fMRI data (Moerel et al., 2013). However, in these approaches the distinct sound objects need to be presented in isolation, using paradigms that require very large numbers of repetitions, over separate imaging sessions, limiting their practical applicability in, for example, characterizing functional abnormalities.

One possible way to address the challenge of defining complex functional brain networks with a limited amount of data is through the analysis of resting-state functional connectivity. At the whole-brain level, this analysis estimates correlations of spontaneous neural activity in the absence of specific tasks or stimuli to investigate the brain’s intrinsic functional organization and its abnormalities under different conditions. Connectivity-based fMRI studies have, for example, shown that brain functional connectomes are highly individual (Finn et al., 2015) and that they can predict individual behavior and cognition (Baldassarre et al., 2012; Godefroy et al., 2022; Kong et al., 2021; Lauren et al., 2021; Rosenberg et al., 2020; Weissberger et al., 2020; W. F. Z. Yang et al., 2021), as well as the results of clinical interventions (Lan et al., 2022; Meinke et al., 2024; Wang et al., 2024). Recent fMRI studies have also investigated the functional connectivity of AC (Hakonen et al., 2025; Luo et al., 2024; Ren et al., 2021). These studies show that AC can be divided into subareas based on its functional connections and that the functional connectivity of AC is individually variable (Hakonen et al., 2025; Luo et al., 2024; Ren et al., 2021) and correlates with various auditory dysfunctions (Kok et al., 2022; Rosemann et al., 2020; Wilson et al., 2022). However, most studies have aimed to quantify functional connectivity between regions as a static measure that is assumed to be constant over time. This may have resulted in an incomplete understanding of AC’s capacity to organize rapidly evolving auditory sequences into coherent percepts.

Studies with fMRI and other modalities (Boly et al., 2007; Eichele et al., 2008) have suggested that there is a repertoire of metastable coactivation patterns that are expressed over time (Bressler & Kelso, 2001; Deco et al., 2011; Rabinovich et al., 2006). The fluctuating patterns of interactions between distributed regions appear to be an intrinsic property of mammalian brain organization, which may facilitate the dynamic integration, coordination, and response to internal and external stimuli that are critical for ongoing cognition and behavior (Büchel & Friston, 2000; Tognoli & Kelso, 2009). A recently developed approach to studying how brain functional connections fluctuate over time is to identify recurring brain coactivation patterns from fMRI data that represent instantaneous network configurations at single time points (Liu et al., 2018). Analysis of single-volume coactivation patterns has been successfully used to investigate large-scale network dynamics at the whole-brain level (Khan et al., 2022; Liu et al., 2018; Marshall et al., 2020; H. Yang et al., 2023; S. P. Zhang et al., 2023). Interestingly, a recent study (L. Zhang et al., 2025) also provided evidence that the brain-wide coactivation patterns observed during hand movements can be identified during resting state. If the processing modes of AC could be similarly identified from the resting state data, it could simplify data acquisition procedures, which is important, especially in clinical applications where patient compliance and scan time are often limited.

**Fig. 1.**
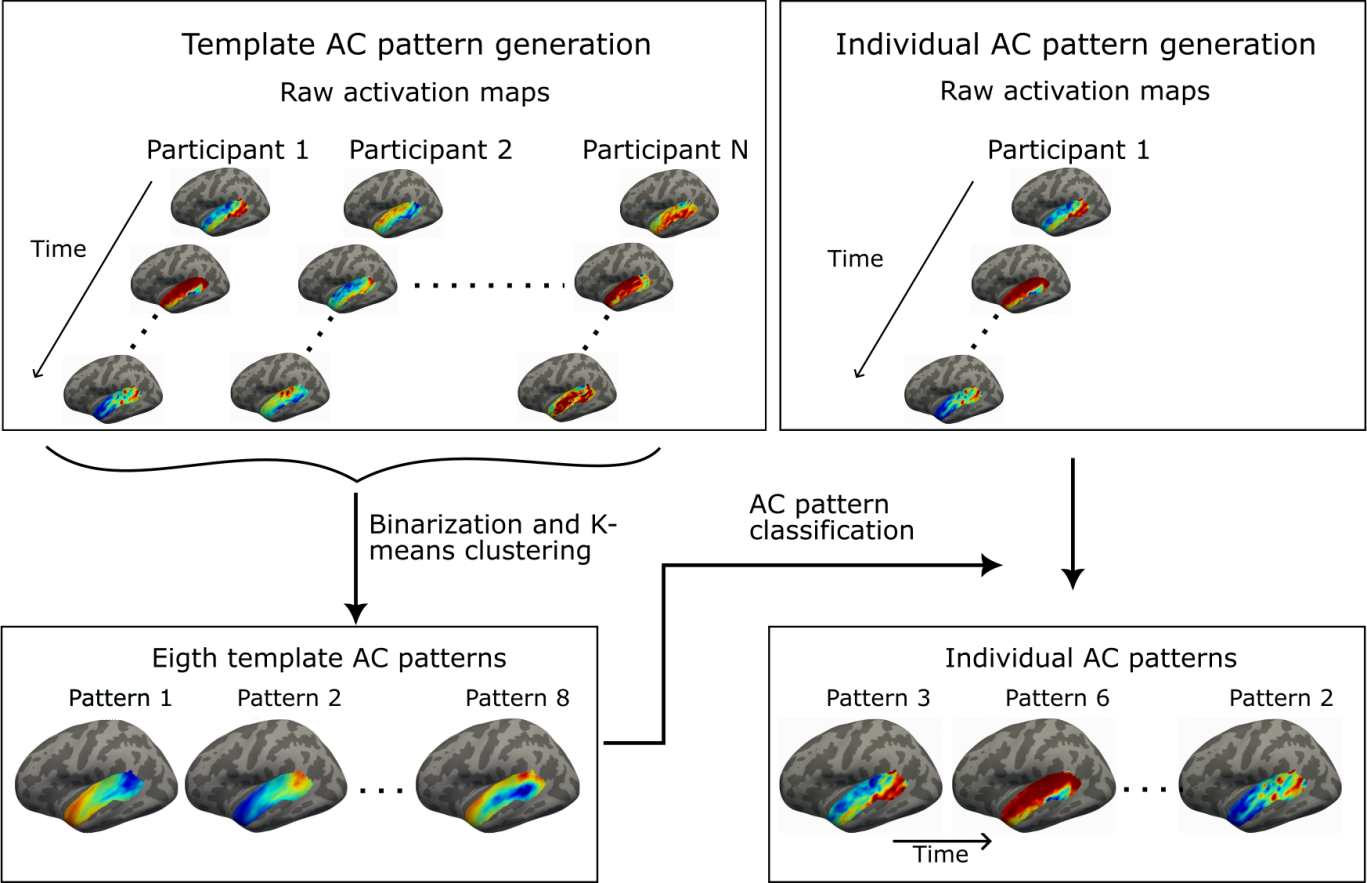
Schematic of the AC pattern generation. The template AC patterns were generated by first binarizing the coactivation maps of each time frame of each participant (i.e., values larger than 0 were set to 1, and values smaller than 0 were set to −1). Thereafter, a k-means clustering algorithm was used to classify the binarized fMRI frames into 8 clusters. Finally, the fMRI frames assigned to each cluster were averaged to create group templates for 8 patterns. The number of patterns was selected based on test-retest reproducibility and visual inspection. At the individual level, each of the fMRI time frames of an individual was classified as the template pattern to which it has the highest spatial similarity.

We used the coactivation pattern analysis (Fig. 1; Peng et al., 2023) to identify metastable “coactivation patterns” in auditory areas of the superior temporal cortices (STC; see, Supplementary Fig. S1 for specific areas) during rest as well as during auditory and audiovisual stimulation. We estimated the AC patterns from high-resolution 7T fMRI measured in our previous study of 30 healthy volunteers, each of which participated in three 2-hour fMRI sessions (termed hereon “MGH-1 mm dataset”; Hakonen et al., 2025). The generalizability of the AC patterns was tested using the Human Connectome Project 7T fMRI dataset (Van Essen et al., 2012). 7T fMRI was used because it offers higher spatial resolution compared to the conventional 3T fMRI without compromising the signal-to-noise ratio (Uğurbil, Adriany, et al., 2003). Additionally, 7T fMRI more accurately reflects the underlying neuronal activity than 3T fMRI, due to the increased contribution of smaller vessels at the higher field strength (Ogawa et al., 1992; Uğurbil, Toth, et al., 2003; Uludağ et al., 2009).

We hypothesized that coactivation patterns similar to those identified at the whole-brain level could also be reliably identified locally within AC. We further anticipated that the AC patterns identified during the resting state would represent fundamental functional processing modes, showing strong spatial similarity to patterns identified during auditory and audiovisual stimulation. We also expected that the AC patterns would synchronize temporally with specific properties of the auditory and audiovisual input. Finally, we hypothesized the AC patterns to be idiosyncratic, reflected by greater variability in coactivation patterns and occurrence rates between individuals than within individuals.

## 2 Methods

### 2.1 Data

#### 2.1.1 MGH-1 mm dataset

##### 2.1.1.1 Participants

The MGH-1 mm dataset consisted of the same 7T fMRI data from 30 healthy volunteers (32.4 ± 10 years, 15 women, all right-handed) that was published in our previous study (Hakonen et al., 2025). Twenty-eight of the participants were native English speakers, and none of them reported hearing impairments, exposure to excessive noise, or the use of cognition-altering medications. All except one of 20 participants who participated in the Hughson-Westlake pure tone procedure had hearing thresholds below 25 dB at 0.125–8 kHz (for one participant, 40-dB threshold at 6 and 8 kHz in the left ear and 35 dB at 8 kHz in the right ear). The study protocol was approved by the Institutional Review Board at Mass General Brigham and carried out in accordance with the guidelines of the declaration of Helsinki. All participants provided a written informed consent prior to the experiments.

##### 2.1.1.2 Experimental setup

The participants completed two resting-state fMRI sessions on two different days. During each session, six 8-minute fMRI runs were conducted (except for one participant, for whom four runs were conducted in the other resting-state session). During the resting-state scans, the participants were presented with a white fixation cross on a black background.

In addition to the resting-state measurements, the participants participated in task-fMRI measurements during which they completed a combined tonotopy and amplitude modulation (AM) rate representation task (2 × 8 minutes) and an audiovisual speech perception task (4 × 11 minutes). Eight participants completed the tasks in two separate days, and the other participants completed them in the same session on the same day. In the task sessions, participants were instructed to attentively perform the given auditory task by indicating their responses using an MRI-compatible response pad. Stimulus presentation was controlled using Presentation software (Neurobehavioral Systems, Berkeley, CA, USA). The tasks were designed to identify the main functional areas of AC in a time-efficient manner.

The aim of the “Tonotopy/AM task” was to localize the frequency tonotopy and AM rate gradients of the AC. Participants were presented with sequences of blocks, each containing seven sounds that varied in both center frequency (for tonotopic mapping) and amplitude modulation (AM) rate (for rate representation mapping). The carrier sounds consisted of octave-wide bands of filtered white noise with center frequencies of 0.12, 0.47, 1.87, or 7.47 kHz. Each of the four carrier signals was amplitude-modulated at either ~4 Hz (slow AM) or ~32 Hz (fast AM). In 85% of the blocks (non-targets), the AM rate varied randomly at seven possible 1/8 octave steps around, and including, the center of the AM rate window (adapted and modified from Norman-Haignere et al., 2019). In the remaining 15% (target blocks), the AM rate remained constant at either 4 or 32 Hz. Each 8-minute run consisted of 64 sound blocks interleaved with silent baseline blocks. The block presentation order was optimized using the automatic event scheduling tool Optseq (FreeSurfer: https://surfer.nmr.mgh.harvard.edu/optseq/), while the order of sounds within each block was randomized using MATLAB’s randperm function. Participants were instructed to fixate on a cross on the screen, ignore changes in carrier frequency, attend to the AM rate, and press a button with their right index finger when they detected a target block with a constant AM rate.

The Audiovisual Speech/Noise task was employed to localize brain regions processing speech and audiovisual information. The participants were presented with 636 audiovisual recordings (159 per run) of a female speaker voicing the words “rain” or “rock” (Ozker et al., 2017, 2018). The auditory component was either acoustically intact or replaced with spectrally matched noise that preserved the original speech’s spectrotemporal power distribution (see Ozker et al., 2017 for stimulus creation details). Likewise, the visual component was either intact or replaced with a blurred version of the original video. These auditory and visual components were combined to form four conditions for each word (“rain” and “rock”): Auditory Clear / Visual Clear, Auditory Clear / Visual Noisy, Auditory Noisy / Visual Clear, Auditory Noisy / Visual Noisy

The stimuli were obtained from: https://openwetware.org/wiki/Beauchamp:Stimuli#Stimuli_from_Ozker_et_al. Participants responded by pressing one button with their right index finger when hearing “rain” and another with their right middle finger when hearing “rock.” The audio intensity was adjusted individually to ensure a comfortable listening level clearly audible above the scanner noise. Visual stimuli and a fixation cross were projected onto a mirror attached to the head coil.

##### 2.1.1.3 fMRI measurements

MRI data were acquired using a 7T whole-body MRI scanner (MAGNETOM Terra, Siemens, Erlangen, Germany) with a custom-built 64-channel receive brain array coil and a single-channel birdcage transmit coil (Mareyam et al., 2020). Each imaging session included: **1)** T1-weighted anatomical images collected using a 0.75-mm isotropic Multi-Echo MEMPRAGE pulse sequence (van der Kouwe et al., 2008; TR = 2,530 ms; TE = 4 echoes with TEs 1.72, 3.53, 5.34 and 7.15 ms; flip angle = 7°; field of view, FoV = 240 × 240 mm^2^; 224 sagittal slices), **2)** blood-oxygenation-level-dependent (BOLD) fMRI data collected using a single-shot 2D simultaneous multi-slice echo planar imaging (EPI) sequence (Setsompop et al., 2012; blipped-CAIPI, acceleration factor in slice-encoding direction: 3; acceleration factor in phase-encoding direction: 4; TR = 2,800 ms; TE = 27.0 ms; 1-mm^3^ isotropic voxels; flip angle = 78°; FoV = 192 × 192 mm^2^; 132 axial slices; phase enc. dir.: anterior→posterior; readout bandwidth = 1,446 Hz/pixel; nominal echo spacing = 0.82 ms; fat suppression), **3)** a short EPI scan (three repetitions) collected with the same parameters, but with the opposite phase-encoding direction polarity (posterior→anterior, PA-EPI), and **4)** a gradient-echo based B_0_ field map (TR = 1,040 ms, TEs = 4.71 ms and 5.73 ms; 1.3-mm^3^ isotropic voxels; flip angle: 75°; FoV: 240 × 240 mm^2^; 120 slices; bandwidth = 303 Hz/pixel). In addition, T2-weighted anatomical images were acquired for 28 out of 30 participants with the T2 SPACE sequence (voxel size = 0.83 × 0.83 × 0.80 mm, TR = 9,000 ms, TE = 269 ms, flip angle = 120°, FoV = 225 × 225 mm^2^, 270 sagittal slices) in one session. The different contrast of T2 images was used to improve pial surface reconstruction. During the fMRI data acquisition, participants’ heart rate and respiration signals were recorded at 400 samples/s using the Siemens photoplethysmogram transducer and respiratory belt.

#### 2.1.2 Human Connectome Project 7T fMRI dataset

The generalizability of the AC coactivation patterns was tested using resting-state 7T fMRI and structural 3T MRI data measured from 184 participants (age range: 22–35, the ages were reported in age bands of 3–4 years, two participants were over 36 years old, 112 women) in the Human Connectome Project (HCP, https://www.humanconnectome.org/hcp-protocols-ya-7t-imaging). The structural data were acquired using a customized Siemens 3T Connectome Skyra scanner with a standard 32-channel Siemens receive head coil. T1-weighted anatomical images were collected using a MPRAGE pulse sequence (accel. factor PE = 2; multi-band accel. factor = 5; TR = 2,400 ms; TE = 2.14 ms; 0.7 mm^2^ isotropic voxels; flip angle = 8°; field of view, FoV = 224× 224 mm^2^; 256 slices). The resting-state data were acquired using a Siemens Magnetom 7T MR scanner with the Nova32 32-channel Siemens receive head coil and an incorporated head-only transmit coil. The total amount of the resting-state data measured in each session was approximately 16 minutes. The participants were instructed to keep their eyes open with a relaxed fixation on a projected cross on a dark background. Oblique axial slices were acquired in a posterior-to-anterior direction in runs 1 and 3 and in an anterior-to-posterior phase encoding direction in runs 2 and 4. The fMRI data were acquired with the gradient-echo EPI sequence (accel. factor PE = 2; multi-band accel. factor = 5, TR = 1,000 ms, TE = 22.2 ms, 1.6 mm^3^ isotropic voxels, flip angle = 45°, FOV = 208 × 208 mm^2^, 85 slices, readout bandwidth = 1,924 Hz/pixel; nominal echo spacing = 0.64 ms).

### 2.2 Preprocessing

#### 2.2.1 MGH-1 mm dataset

The MGH-1 mm dataset was preprocessed using Freesurfer version 7.1 and our custom MATLAB and bash scripts. Preprocessing of the structural T1 and T2 images of started with bias field correction using SPM12 (http://www.fil.ion.ucl.ac.uk/spm/, spm_preproc_run.m; bias full-width at half-maximum, FWHM: 18 mm, sampling distance: 2 mm, bias regularization: 1e-4) and custom MATLAB scripts. Thereafter, the cortical surface reconstructions of the interfaces between gray and white matter and pial surface were generated using the recon-all function of FreeSurfer 7.1 (Fischl, 2012) with an extension for submillimeter 7T data (Zaretskaya et al., 2018). To enhance the quality of the cortical surface reconstruction, both a T2-weighted image and an average over 3–4 T1-weighted images from different sessions were used in the reconstruction. For intracortical smoothing, nine intermediate cortical gray matter surfaces were generated at fixed intervals between the white and pial surfaces produced by recon-all. The reconstructed surfaces were visually inspected for quality assurance, and any segmentation errors in the pial or white matter boundaries were manually corrected using the Recon Edit tool in Freeview.

The functional data were first corrected for slice timing and head motion. The head motions were corrected by aligning individual volumes to the middle volume of each run. Dewarping was applied to address geometric distortions caused by static magnetic field inhomogeneities. The distortion field used for dewarping was estimated from the fMRI runs and a phase-encoding reversed (posterior-anterior, PA) run, which has distortions equal and opposite to those in the fMRI (anterior-posterior, AP) runs. Susceptibility-induced off-resonance field maps were calculated using the method described by Andersson et al. (2003), as implemented in the FMRIB Software Library (FSL; version 5.0.7) tools topup and applytopup (K. R. Smith et al., 2004). For four participants who lacked the PA scan, the distortion field was estimated using a B0 field map with FreeSurfer’s epidewarp tool. Artifacts caused by cardiac and respiratory cycles were reduced using the RETROspective Image CORrection (RETROICOR) algorithm (Glover et al., 2000) (third-order heart rate, respiratory, and interaction terms). For three participants only respiratory signals were used because their heart rate data were missing. For five participants with no physiological recordings, RETROICOR was not applied. Functional images were co-registered to anatomical images using boundary-based registration (Greve & Fischl, 2009) via preproc-sess in FreeSurfer 7.1 and then further transformed to FreeSurfer cortical template (fsaverage6, 40 962 vertices per hemisphere). Spatial smoothing was performed using an intracortical surface-based approach. This method restricts smoothing to cortical gray matter, reducing signal contamination from white matter and cerebrospinal fluid (Andrade et al., 2001; Blazejewska et al., 2019; Jo et al., 2007; Kiebel et al., 2000). The fMRI time series were first resampled onto 11 intermediate surfaces between the white and pial surfaces using trilinear interpolation. These surfaces were then used to generate a single smoothed intracortical surface using a smoothing kernel that operated tangentially across a 7th-order vertex neighborhood and radially across 10 intracortical surfaces. The outermost cortical surface was excluded to minimize partial volume effects from cerebrospinal fluid. Following smoothing, the data were bandpass filtered between 0.01–0.08 Hz, and signals from white matter, cerebrospinal fluid, and non-brain voxels were regressed out using general linear model analysis (GLM, FreeSurfer mri_glmfit). The resulting centered residuals (i.e., zero mean) were used in the analyses.

#### 2.2.2 Human Connectome Project 7T fMRI dataset

We downloaded the HCP structural MRI data that had already been processed with the PreFreeSurfer pipeline (Glasser et al., 2013). This included gradient nonlinearity distortion correction, coregistration and averaging of the T1 run and T2 run, alignment along the anterior and posterior commissure (AC–PC) axis, brain extraction, field map distortion correction, registration of T2 to T1, bias field correction, and MNI nonlinear volume registration. We generated the cortical reconstructions from these data for each participant using the recon-all function of FreeSurfer 7.1 (Fischl, 2012). The T2 anatomical data were used to improve the outcome for pial surface reconstruction in the recon all algorithm. The initial preprocessing included motion correction using 6-DOF FLIRT, gradient nonlinearity distortion correction, and EPI to T1w anatomical registration (Glasser et al., 2013). The original EPI frames were resampled to the atlas space via one-step spline resampling that included all transforms (motion correction, EPI distortion correction, EPI to T1w with FLIRT BBR, fine-tuning of EPI to T1w with bbregister, nonlinear T1w to MNI). The bias field estimated from the structural data was also removed from the fMRI data. The fMRI data was masked and normalized to a 4D whole brain mean intensity of 10,000. We resampled these preprocessed volumes into fsaverage6 surface, spatially smoothed them with a 3D 6-mm full-width at half-maximum Gaussian kernel, band-pass filtered the data between 0.01–0.08 Hz, and regressed out head-motion parameters and ventricular and white-matter signals using GLM. The residual values (i.e., zero mean) from the GLM were used in the analyses.

### 2.3 Single-frame dynamic coactivation pattern analysis

Dynamic function of AC was investigated using the single-frame dynamic coactivation pattern analysis approach. The coactivation pattern analysis estimates functional connectivity by identifying coactivation patterns from single timeframes of fMRI data, rather than calculating correlations between signals of regions over time windows, as has been done in the traditional sliding window methods. The assumption is that the areas that are spontaneously coactivated are also functionally connected. The use of single timeframes allows for the detection of transient functional network interactions at finer temporal resolution and offers a more accurate assessment of their temporal dependencies. Furthermore, unlike sliding window methods that rely on second-order statistics such as temporal correlations, single-frame analyses are not influenced by “sampling variability” (Laumann et al., 2017).

The coactivation patterns were determined within the cortical area defined by combining the transverse temporal, superior temporal, and banks of the superior temporal sulcus (bankssts) labels from the Desikan-Killiany Atlas (Desikan et al., 2006). The analysis started with the generation of group-level template coactivation patterns, which were thereafter used to estimate individual-specific patterns (Peng et al., 2023). To create the template patterns, each of the fMRI frames (i.e., each single timepoint of the fMRI data right after preprocessing) was first binarized so that positive BOLD-signal values were set to 1 and negative values to −1. The binarized fMRI frames of all participants were clustered into eight clusters using the k-means algorithm. Finally, the binarized frames were averaged across participants in each cluster. The resulting eight averaged maps were used as group templates of AC patterns.

The individual-specific AC patterns were estimated by comparing each of the fMRI time frames to the template patterns and assigning it to the pattern with the highest spatial similarity (cosine similarity, MATLAB, pdist function). The time frames classified as the same AC pattern were averaged to create an individual-level AC pattern map. At the individual level, the frames were not binarized. The pattern occurrence rates were determined as the number of time frames assigned into the same AC pattern divided by the total number of time frames. In the visualization, we used coactivation patterns, each of which was bilaterally normalized between 0 and 1. Our cross-dataset comparisons were, thus, intended to assess spatial similarity rather than relative coactivation strength.

To estimate the optimal number of clusters or patterns, we assessed test–retest reproducibility between resting-state sessions of the MGH-1 mm dataset for even cluster numbers ranging from 2 to 20. Even numbers were chosen because the coactivation patterns were represented in pairs with opposite spatial topographies. Individual-level coactivation patterns were derived separately for each resting-state session using templates generated from both sessions. Reproducibility was quantified for each participant by computing Spearman correlations between the average coactivation patterns across sessions. We further examined pattern similarity as a function of cluster number by calculating the mean Spearman correlation between pattern topographies for each cluster solution. In addition, the optimal cluster number was estimated using the Elbow method, Silhouette score (Rousseeuw, 1987), Davies–Bouldin index (Davies & Bouldin, 2009), and Calinski–Harabasz index (Caliński & Harabasz, 1974). To minimize the effect of motion artifacts on the templates, the templates were determined only using the participants with a mean and maximum head motion (framewise displacement) under 0.1 and 0.5 mm, respectively. Of the total 60 sessions (two sessions for each of the 30 participants), 55 fulfilled this criterion.

Binarization was used in the template pattern generation to follow the procedures of Peng et al. (2023), which used binarized maps in their whole-brain coactivation analysis. An alternative approach could have been to select certain percentage of the strongest activations and deactivations, similarly, as have commonly been done for correlation matrices when estimating functional connectivity with correlations between brain signals, which could in some cases help avoid biases due to physiological noise. Another simpler approach is to use nonbinarized timeframes with continuous values has also produced reliable results at least in the brain-wide analyses (Li et al., 2024; H. Yang et al., 2021, 2023).

We also attempted to weight the raw activation maps with parcellation confidence derived from group-level functional connectivity-based AC parcellations consisting of 4 and 6 functional networks (Hakonen et al., 2025), correspondingly as was done for the whole-cortex analysis in the previous study (Peng et al., 2023). The aim of this approach is to reduce the effect of noise and preserve the inherent network structure of the coactivated data. In the AC parcellations the networks were defined to be sets of vertices with similar profiles of functional connectivity. The connectivity profile of a vertex was its functional coupling to the other cortical vertices. The parcellation confidence indicates the probability that the vertex belongs to its assigned network. The confidence value for each vertex was defined as correlation of the vertex with the other vertices in the same network compared with the vertices belonging to the network with which the vertex had the second highest correlation. The confidence values ranged between -1 and 1, and the larger value indicated higher confidence of a vertex to belong to its assigned network. However, weighting the raw activation maps with parcellation confidence resulted in spatial topography maps that contained no clear, continuous, larger activated/deactivated networks. This may be because the networks in the parcellations were much smaller than the ones used for the whole brain (Peng et al., 2023). The group-level networks may not have aligned with the individual-level networks because of the interindividual variability. Therefore, we decided not to weight the raw activation maps with the parcellation confidence in contrast to the study of Peng et al. (2023). Our approach is consistent with other previous studies that have analyzed coactivation patterns without weighting the data frames with the parcellation confidence (Gutierrez-Barragan et al., 2019; Huang et al., 2020; Janes et al., 2020; H. Yang et al., 2023).

### 2.4 Reproducibility and generalizability of AC patterns

The reproducibility of the AC patterns was estimated by deriving the template and individual AC patterns separately from the first and second resting-state sessions of the MGH-1 mm dataset. Both group average and individual-level coactivation map topographies were compared between the two sessions with Spearman correlation and the occurrence rates with Pearson correlation. At the individual level, the AC pattern similarity within and between participants was compared using the Wilcoxon rank sum test. The correlation values were transformed into z-scores with Fisher’s z transformation for the Wilcoxon rank sum test.

We also tested whether the AC patterns identified using the templates determined from independent dataset produce similar results than templates derived from the same dataset. To this end, the template AC patterns were derived separately from the resting-state MGH-1 mm data and HCP data. Thereafter, the individual-level AC patterns were determined from the MGH-1 mm data using the MGH-1 mm and HCP templates. Finally, the group-average AC pattern topographies and occurrence rates were derived using MGH-1 mm templates and compared to the ones derived using HCP templates.

The generalizability of the AC patterns was tested by deriving both the AC template patterns and individual-level patterns within MGH-1 mm and HCP datasets and comparing the group-average results. To estimate the generalizability of the AC patterns between resting state and auditory stimulation, the template AC patterns were derived separately from the resting-state and task MGH-1 mm data. Thereafter, the individual-level AC patterns were determined from the MGH-1 mm data using the resting-state and task templates, and the group-averages over them were compared to each other.

In all reproducibility and generalizability analyses, the pairing of patterns between conditions was optimized using the Hungarian algorithm (Cao, 2025; Kuhn, 1955). Furthermore, the templates and individual-level patterns were derived independently within each dataset. Cross-dataset comparisons were performed only after all within-dataset analyses, ensuring that no data were reused for both pattern definition and evaluation.

To evaluate the potential influence of spatial dependencies or autocorrelation on our results, we generated eight coactivation patterns by averaging randomly selected frames from the second resting-state session of the MGH-1 mm dataset. Each random pattern was created by averaging a number of frames proportional to the occurrence rate of its corresponding actual pattern. We then calculated the correlation between each actual coactivation pattern derived from the first resting state session and its corresponding random pattern from the second session. This process was repeated 1,000 times, and the maximum correlation across patterns was used to construct the null distribution. Finally, the correlations between the actual patterns derived from the first and second resting-state sessions were compared against the null distribution.

### 2.5 Interindividual variability of AC patterns

Group-level templates of the coactivation patterns can be expected to represent tendencies that reflect typical AC function. When each of the participant’s timeframes is classified to the best-matching template pattern and the timeframes classified into the same templates are averaged, the resulting individual-level averages should retain participant-specific features in addition to the features that reflect typical brain. Therefore, this approach also allows for studying interindividual variability in the spatial topographies of the identified brain states. Moreover, interindividual variability can be studied not only in the spatial topographies but also in the occurrence rates of these patterns, which may differ between individuals even when their spatial topographies are broadly similar.

The interindividual variability in the coactivation patterns and their occurrence rates were estimated with pairwise comparisons between participants using resting-state MGH-1 mm data. The similarity of the spatial topographies was estimated using the Spearman correlation and the similarity of the occurrence rates with the Pearson correlation. The correlation values were transformed into z-scores with Fisher’s z-transformation. Thereafter, the similarity between participants was compared to the similarity between two resting-state sessions within participants using the Wilcoxon rank sum test. Comparison to the within-participant similarity allowed us to estimate what part of the interindividual variability could be explained with noise.

The Mantel test (Glerean et al., 2016) was further used to test whether interindividual variability in the pattern spatial topographies reflects interindividual variability in cortical thickness or curvature. The Mantel test is a statistical method used to estimate correlation between similarity / dissimilarity matrices. It is particularly useful for understanding whether relationships in one dataset correspond to the relationships in another. The thickness and curvature maps for each participant were created using the FreeSurfer recon-all pipeline, and the AC area used in the AC patterns was extracted from the thickness and curvature maps (see our previous study, Hakonen et al., 2025, for the correspondence between the AC curvature maps and the position and shape of the HG). Similarity matrices for cortical curvature and thickness were created by calculating pairwise Spearman correlations between participants using vectors representing their AC thickness and curvature, respectively. For each of the eight AC pattern topographies, we created a similarity matrix by calculating pairwise Spearman correlations between vectors representing participant-specific pattern topographies. Finally, an average similarity matrix was computed over eight pattern-specific similarity matrices. In the Mantel test, the Spearman correlation was computed between the upper triangle elements of the AC pattern topography similarity and thickness similarity matrix, as well as between AC pattern topography and curvature similarity matrix. To assess statistical significance, null distributions were generated separately for each comparison by performing 10,000 random permutations of the rows and columns of the AC pattern topography similarity matrix and recalculating the correlation for each permutation. The p-value was computed as the percentage of the null-distribution values larger than the actual correlation value. The threshold of statistical significance was set at 0.05.

### 2.6 Correlation between the occurrences of AC coactivation patterns and task contrast regressors

To test whether AC coactivation patterns could be associated with AC function, we calculated correlations between the AC pattern occurrences and task contrast regressors. The vectors indicating AC pattern occurrences were created by classifying each time frame of task fMRI data of each individual into one of the eight AC patterns. The time frames were classified by assigning them to the group template pattern with the highest spatial similarity to it (cosine similarity, MATLAB, pdist function). The group template patterns were derived from the resting-state data. The analysis was conducted using the group average over the individual-specific occurrence rate vectors that represented the proportion of frames assigned to each AC pattern.

We generated regressors for 11 task contrasts. For the Audiovisual Speech / Noise task, the contrast regressors were computed across the Auditory Clear / Visual Clear (AcVc), Auditory Clear / Auditory Noisy (AcVn), Auditory Noisy / Visual Clear (AnVc), and Auditory Noisy / Visual Noisy (AnVn) stimuli. In the main effect of the Audiovisual vs. fixation cross, we contrasted all possible audiovisual combinations with the fixation cross. In the main effect of Speech vs. Noise, all possible audiovisual combinations were contrasted with clear auditory signal with those with noisy auditory signal, that is, ((AcVc+AcVn) − (AnVc+AnVn)) / 2. In the main effect of Lip motion vs. Noise, all audiovisual combinations with clear visual signal were contrasted with those with blurred vision, that is, ((AcVc+AnVc) − (AcVn+AnVn)) / 2. We also computed an audiovisual interaction to identify the cortical areas where visual information of lip motion has the strongest effect on processing speech sounds when the auditory signal is noisy. This contrast was, thus, defined as (AnVc-AnVn) − (AcVc-AcVn). Additionally, we computed an audiovisual interaction for identifying cortical areas where auditory information has the strongest influence on the processing of visual information when the video clip is blurred. This contrast was defined as (AcVc-AcVn) − (AnVc-AnVn). Furthermore, we generated regressors indicating the following contrasts: AnVcAnVn, AcVnAnVn, and AcVcAnVc. From the Tonotopy/AM task, the following contrasts were selected: 1) auditory stimulation vs. baseline, 2) high (1.87 or 7.47 kHz) vs. low (0.12 or 0.47 kHz) carrier frequencies, and 3) slow (4 cycles/s) vs. fast (32 cycles/s) amplitude modulation.

Pearson correlation was computed between the occurrence rate vectors and each of the task contrast regressors using MATLAB R2023a. Thereafter, the correlation values were transformed into z-values by using Fisher’s transformation and further to z-scores by dividing the z-values with the standard error of the z-values. The significance values for the z-scores were determined from the z-distribution and corrected for multiple comparisons with the Benjamini-Hochberg procedure (Benjamini & Hochberg, 1995; Groppe, 2024). Only results exceeding the significance threshold of 0.05 were reported.

## 3 Results

### 3.1 Coactivity patterns of AC are reproducible within dataset and generalizable between datasets

**Fig. 2.**
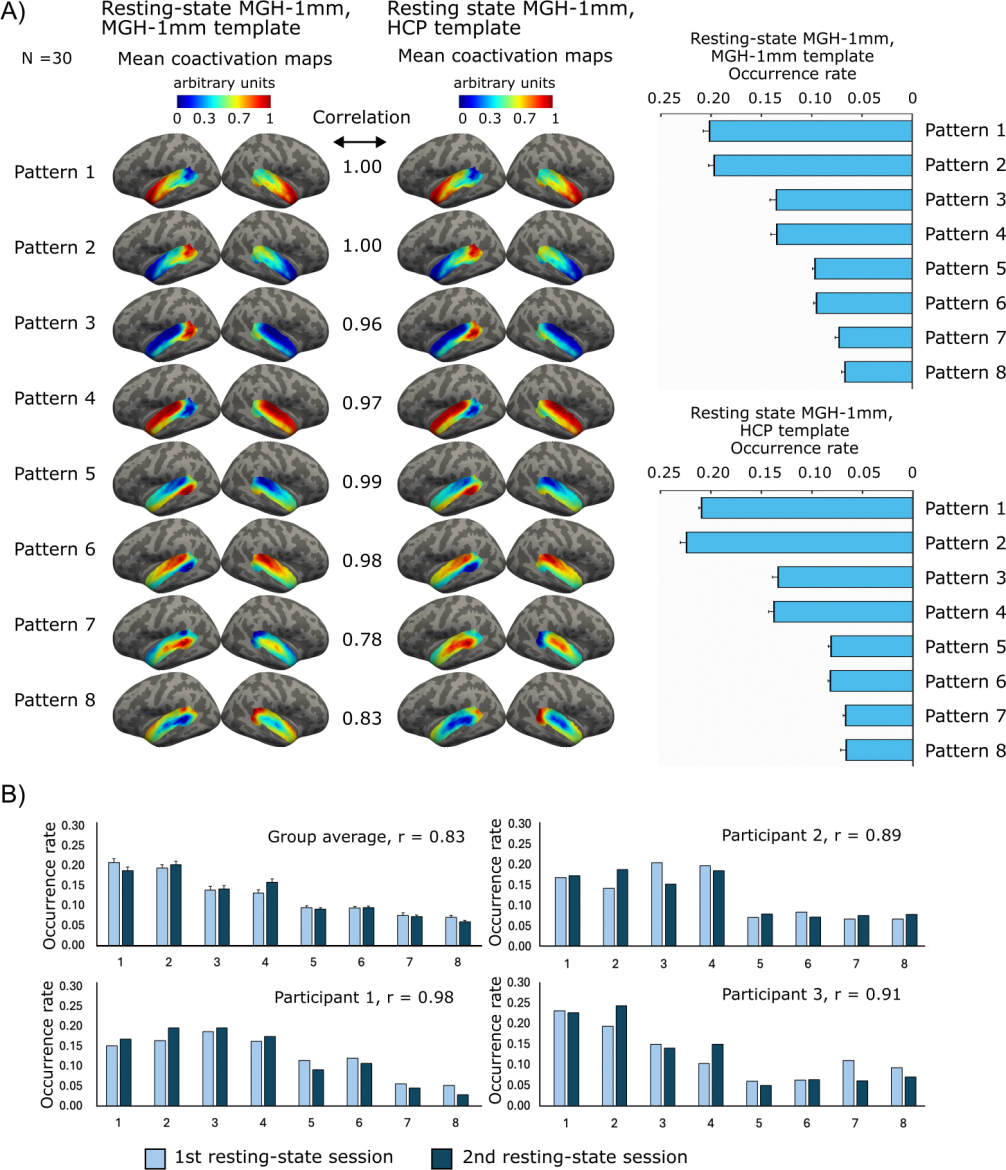
Properties of the AC coactivation patterns. (A) The mean coactivation maps and occurrence rates of the AC patterns computed from the resting-state fMRI data of 30 participants (MGH-1 mm data). The maps were calculated using either the group templates determined from the same MGH-1 mm data or the templates determined from the HCP data. The correlation values indicate Spearman correlation between the corresponding group-average patterns of the two datasets. All correlation values were statistically significant (p < 0.001 for all patterns). (B) AC pattern occurrence rates at the first and second resting-state sessions for group average and three representative participants. Error bars indicate the standard error of the mean (SEM). The group average correlation of 0.83 indicates an average over individual correlations.

Figure 2A shows averages of individual-level coactivation patterns for the eight-pattern solution estimated from 30 MGH-1 mm participants (two fMRI sessions per participant, 48 minutes resting-state data in each session). The anatomical subdivisions of the auditory cortex as defined by the Desikan atlas, are overlaid on the patterns in Supplementary Figure S1. The individual patterns were identified using the group templates derived either from the same MGH-1 mm resting-state data or from an independent resting-state 7T dataset comprising 177 HCP participants. The corresponding group-level coactivation maps obtained using these two template sets were highly similar (Spearman correlation = 0.94 ± 0.09; all patterns p < 0.001; Fig. 2A), indicating that templates derived from a fully independent dataset yield consistent results with those derived from the same dataset.

Different methods for estimating an optimal number of clusters produced inconsistent results, and none identified a single clear solution (Supplementary Fig. S2), consistent with previous co-activation pattern studies (Huang et al., 2020; Karahanoğlu & Van De Ville, 2015; Liu & Duyn, 2013; Liu et al., 2013; H. Yang et al., 2021). The test-retest reliability decreased monotonically beyond four clusters, the Silhouette Score showed an elbow at six clusters, and the cluster similarity exhibited a local minimum at eight clusters. It has also been proposed that these algorithms may underestimate the number of clusters because pattern dissimilarities are likely skewed, with some patterns being much more similar to each other than to the rest, making clear boundaries difficult to define (Liu et al., 2013). In addition, noise in the fMRI volumes further blurs cluster separations. Therefore, we selected 8-cluster solution for further analyses. The 4-, 6-, 10- and 12-pattern coactivation profiles are also shown in the Supplementary Figure S3.

The test-retest reproducibility of the AC patterns for the eight-pattern solution was tested by determining both the group template patterns and corresponding coactivation maps and occurrence rates separately from the first and second resting-state fMRI sessions of the MGH-1 mm dataset. At the group level, the results were highly reproducible, as indicated by a Pearson correlation of 0.96 (p < 0.001; Supplementary Fig. S4) between the mean occurrence rates of the group-level coactivation patterns derived from the two sessions. An average Spearman correlation was 0.96 ± 0.02 (p < 0.001 for all patterns) between the group coactivation maps of the corresponding patterns. The significance values were obtained from the Student’s t distribution for Pearson correlation and from the exact permutation distributions for the Spearman correlation (Matlab corr function). The spatial similarity of the activation maps was estimated with Spearman correlation because it estimates if the regions are activated in a similar pattern independently of the exact amplitudes. The results were also relatively reproducible at the individual level, the average Pearson correlation being 0.83 ± 0.04 between the mean occurrence rates. The average Spearman correlation between the coactivation patterns was 0.77 ± 0.02 (patterns: 1: 0.76 ± 0.02, 2: 0.77 ± 0.02, 3: 0.83 ± 0.01, 4: 0.82 ± 0.01, 5: 0.80 ± 0.01, 6: 0.81 ± 0.01, 7: 0.70 ± 0.03, 8: 68 ± 0.03, p < 0.001 for all AC patterns). The occurrence rates at the first and second resting-state sessions for the group average and three representative participants are shown in Figure 2B.

To assess the potential impact of spatial dependencies or autocorrelation on the results, we generated coactivation patterns by averaging randomly selected frames from the second resting-state session. Eight patterns were created, each representing an average of frames proportional to the occurrence rate of one of the actual patterns. Subsequently, we calculated the correlation between each actual coactivation pattern derived from the first resting-state session and the corresponding random pattern generated from the second resting-state session. This procedure was repeated 1,000 times, and the maximum correlation over the patterns was saved in the null distribution. However, the maximum correlation in the null distribution was 0.41, which is smaller than any of the actual correlations. Thus, the correlations between the actual states cannot be explained with autocorrelation.

Generalizability of the AC patterns was tested by computing both individual patterns and group templates within MGH-1 mm and HCP datasets (Supplementary Fig. S5). This resulted in a correlation of 0.91 (p < 0.002) between the group-level occurrence rates and a correlation of 0.75 between the corresponding coactivation patterns.

Each pattern had an opposite pattern in which the same network was deactivated (Fig. 2A: patterns 1 and 2; 3 and 4; 5 and 6; and 7 and 8). Thus, eight patterns represented the activation or deactivation of four networks. There were no differences in the occurrence rates between the patterns and their opposite patterns (p = 0.19–1, pairwise Wilcoxon signed rank test, corrected for multiple comparisons), except for the patterns 7 and 8 (p < 0.004, pairwise Wilcoxon signed rank test, corrected for multiple comparisons). However, the difference between patterns 7 and 8 was very small: 7.3% of the frames were classified as Pattern 7 and 7.0% as Pattern 8. The occurrence rates were different between the patterns and all other patterns than their opposite patterns (p < 0.001, pairwise Wilcoxon signed-rank test, corrected for multiple comparisons).

### 3.2 Occurrence rates and spatial topographies of AC coactivation patterns are more consistent within than between individuals

To investigate the uniqueness of the AC patterns for each individual, the pattern occurrence rates and coactivation patterns were compared between all participants using the resting-state sessions of MGH-1 mm data. The similarity between participants was further compared to the similarity between the resting-state sessions within participants to estimate which part of the interindividual variability could be explained by the noise-related variability between sessions. The group templates were determined using both resting-state sessions, and the individual-level AC patterns were derived separately from the sessions.

**Fig. 3.**
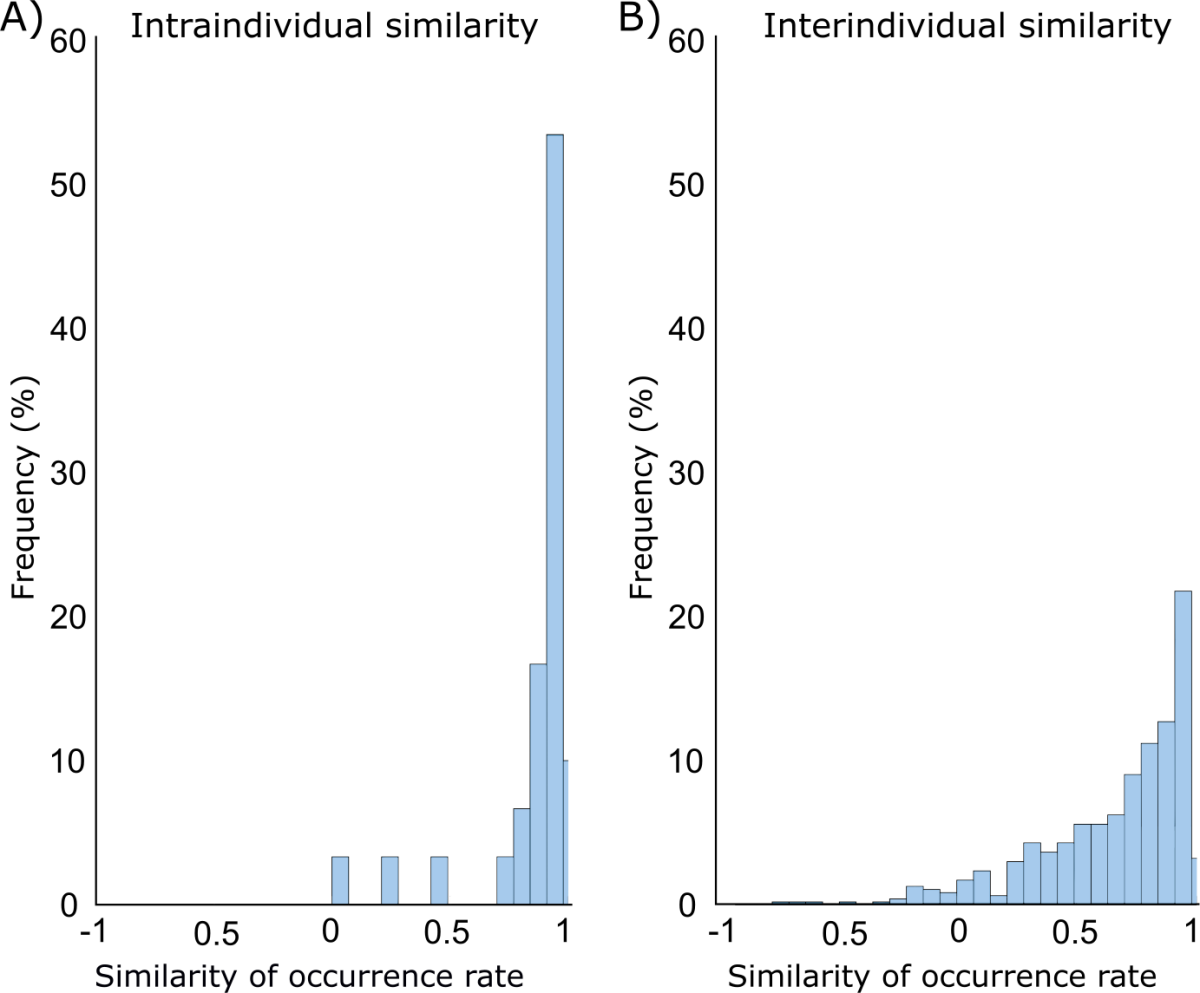
Interindividual variability of AC brain patterns. (A) The frequency distribution illustrates the similarity of AC pattern occurrence rates between the first and second resting-state sessions within participants. (B) The frequency distribution illustrates the similarity of AC pattern occurrence rates between participants.

The mean similarity of the occurrence rates for the eight AC patterns between participants was 0.66 ± 0.32 (i.e., interindividual variability of 0.34). Within participants, the mean similarity was 0.86 ± 0.22, indicating relatively high reproducibility. The occurrence rates were more similar within than between participants (z = 4.8, ranksum = 11064, p < 0.001; Fig. 3) suggesting that observed interindividual differences in connectivity reflect stable, subject-specific features rather than measurement noise. The same was true for the AC coactivation topographies, for which the between-participant similarity was 0.58 ± 0.08 and within-participant similarity 0.79 ± 0.06 (z = 8.4, ranksum = 13803, p < 0.001).

The surface-based anatomical normalization methods used in this study (Fischl et al., 1999) have shown to be less accurate in the areas that are not closely related to major sulci and gyri (Frost & Goebel, 2013; Gulban et al., 2020). Therefore, individual variability of the spatial topographies of the coactivation maps may have been affected by macroanatomical variability between participants. To examine the potential influence of macroanatomy on our results, we performed a Mantel test to assess whether between-participant similarity in activation topographies correlates with between-participant similarity in AC folding patterns or cortical thickness. The Mantel test indicated no significant correlation in the similarity between coactivation topography and macroanatomy within the AC (curvature: r = −0.04, p = 0.62; thickness: r = −0.14, p = 0.88). This suggests that individual differences in coactivation maps cannot be attributed solely to anatomical variability.

### 3.3 AC coactivation patterns share similarities during resting-state and auditory stimulation

**Fig. 4.**
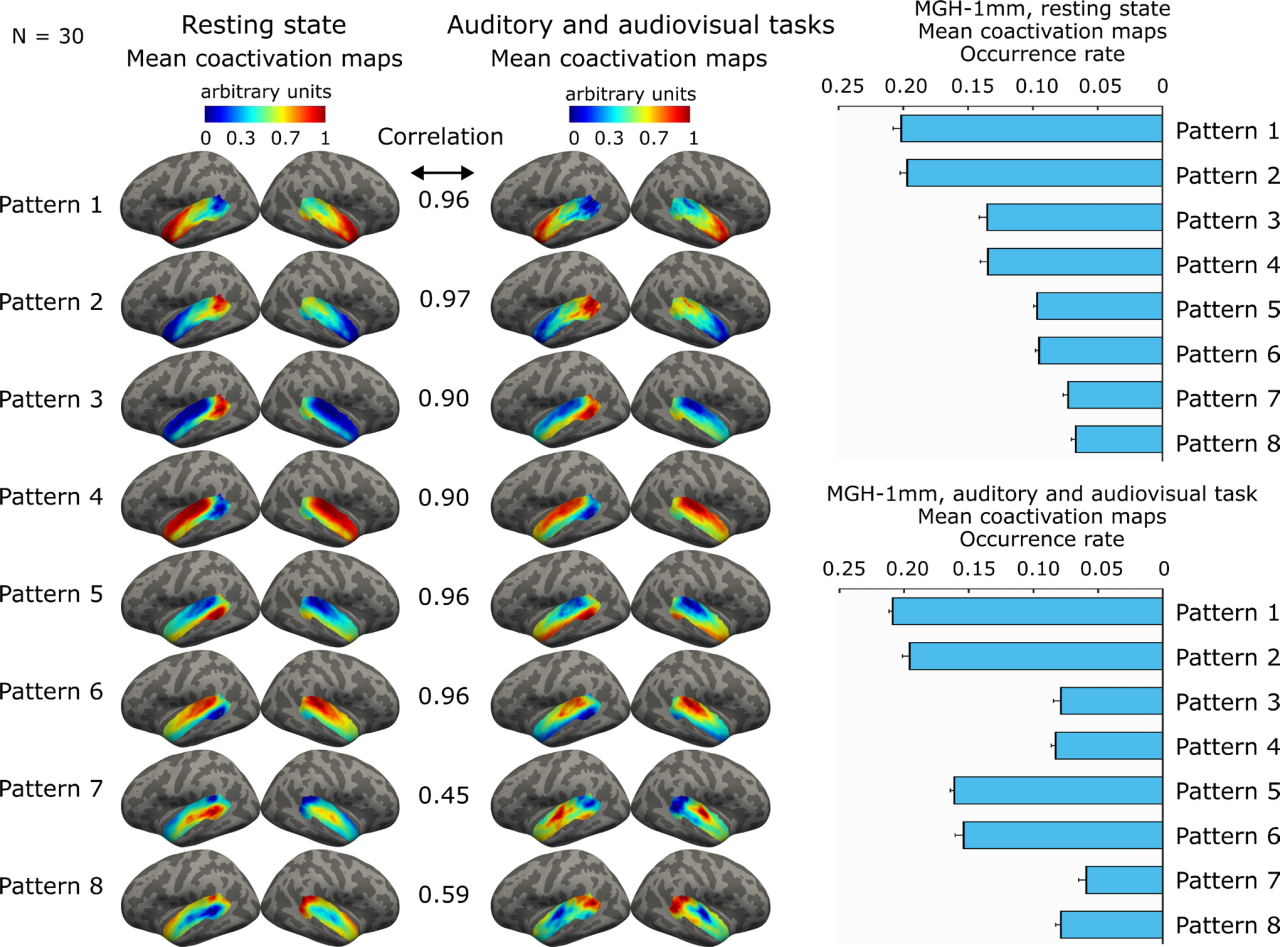
Comparison of occurrence rates and coactivation maps between AC patterns derived from the resting and auditory task fMRI data. The correlation values indicate a correlation between group-average coactivation maps. The resting-state maps were determined using the templates derived from the resting-state data and the task maps using the templates derived from the task data. The task data are ordered based on their spatial correlation with the maps derived from the resting-state data. Error bars indicate the standard error of the mean (SEM).

We hypothesized that intrinsic and task-evoked AC coactivation patterns share common network configurations. To test this hypothesis, we determined the template patterns and individual-level coactivation maps from the data measured during auditory and audiovisual tasks and compared the results to the individual coactivation maps derived from the resting-state data using resting-state group templates (Fig. 4). The average Spearman correlation between the mean AC coactivation maps derived using these approaches was 0.84 ± 0.20, indicating similarities between the coactivation maps (Fig. 4). Especially the first six patterns were highly similar between the approaches (mean correlation 0.94 ± 0.03). The order of the occurrence rates of the pattern pairs 3&4 and 5&6 was opposite between the two approaches. The occurrence rates of pattern pairs 3&4 and 7&8 were closer to each other when the data was measured during auditory stimulation. The differences in the occurrence rates may reflect the engagement of specific networks during the processing of the auditory information.

We also investigated the similarity between the AC patterns derived from the resting-state and task data at the individual level. At the individual level, the correlation of the spatial coactivation maps between task and resting-state data was 0.71 ± 0.07 (p < 0.001, z = 4.8, signed rank = 465; Pattern 1: 0.67 ± 0.02; Pattern 2: 0.67 ± 0.02; Pattern 3: 0.73 ± 0.02; Pattern 4: 0.75 ± 0.02; Pattern 5: 0.73 ± 0.01; Pattern 6: 0.73 ± 0.01; Pattern 7: 0.61 ± 0.02; Pattern 8: 0.60 ± 0.03).

### 3.4 Occurrences of AC coactivation patterns correlate with auditory and audiovisual stimulation

**Fig. 5.**
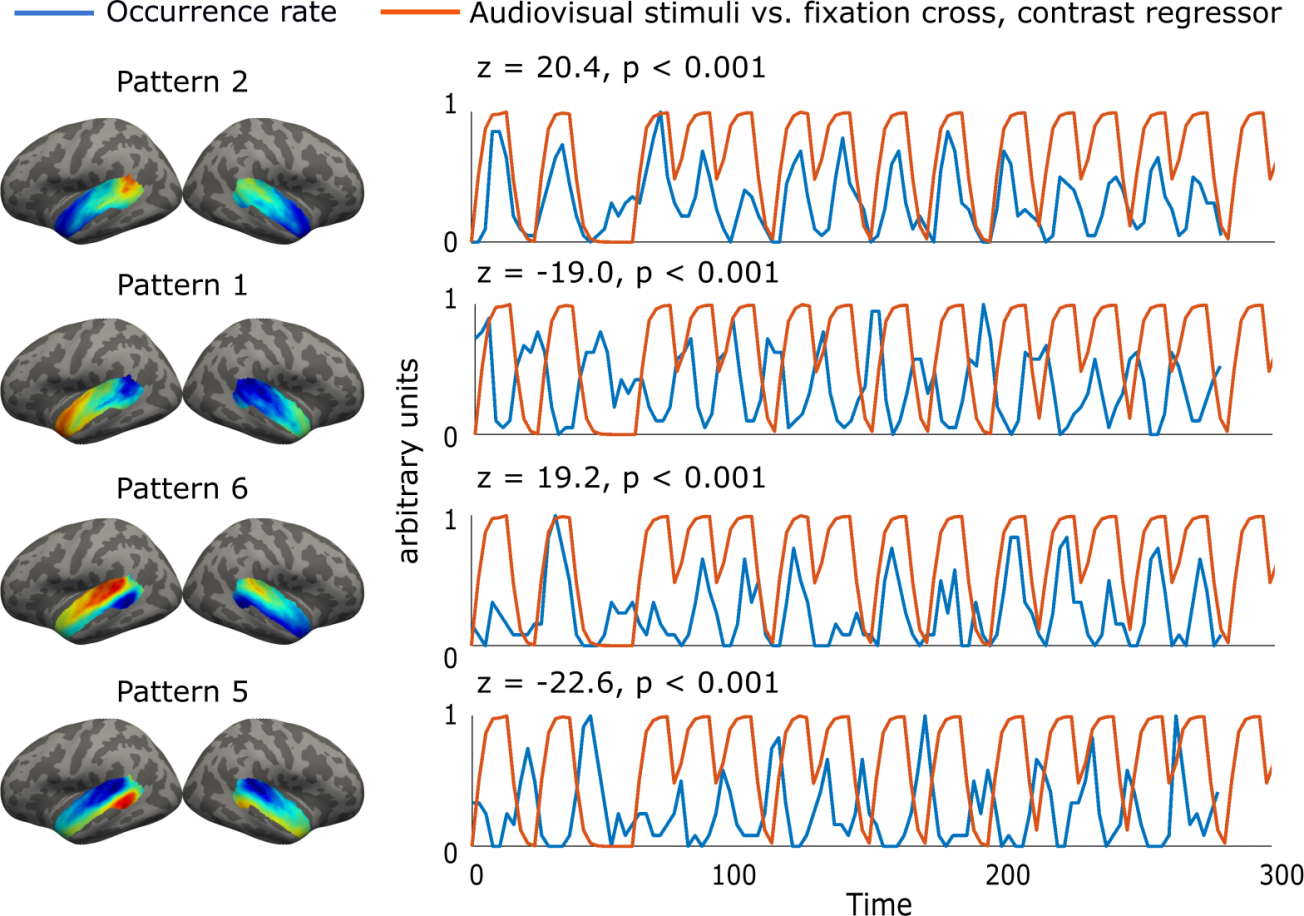
Correlation between AC coactivation patterns and the regressor representing the contrast between audiovisual stimulation and resting state. Patterns 2 and 6 showed positive correlations with the regressor, whereas their corresponding opposite coactivation patterns (patterns 1 and 5) showed negative correlations. The occurrence rates represent the proportion of frames assigned to each AC pattern across all individuals at each timepoint.

To study if the coactivation patterns derived from the resting-state fMRI could be associated with the AC function, we calculated the correlation between the group-averaged occurrence rates of the AC patterns and 11 auditory and audiovisual task contrast regressors (Fig. 5). For this analysis, the occurrence rates were determined from the task fMRI data using the pattern templates derived from the resting-state data.

Each coactivation pattern correlated positively or negatively with 2–9 task contrast regressors (Fig. 5; Table 1). If the pattern correlated positively with a given contrast, its opposite pattern correlated negatively or not significantly with the same contrast. Four AC patterns (2, 4, 6, and 7) showed positive correlations with 4–8 task contrasts. Their opposite patterns (1, 3, 5, and 8) correlated negatively with 2–5 task contrasts and positively with only 1–3 contrasts or not with any contrast.

**Table 1.** Z-values of the correlations between the task contrast regressors and coactivation pattern occurrence rates.

	AC coactivation patterns							
Contrasts	1	2	3	4	5	6	7	8
Aud vs. base	-5.97***	7.12***	-1.09	4.45***	-10.1***	6.99***	2.09	-1.16
Hi vs. low freq.	-0.18	2.75*	-0.02	-2.12	-0.43	-0.45	-2.17	1.95
Slow vs. fast AM	-4.51***	2.81*	-2.06	-1.45	-1.29	4.37***	3.40**	-3.39**
AV vs. fix	-19.0***	20.4***	-3.73**	4.49***	-22.6***	19.2***	-2.10	2.78*
Speech vs. noise	-0.30	1.31	-6.58***	2.23*	-0.55	3.18**	4.41***	-8.02***
Clear vs. blurred	-1.92	2.61*	3.37**	-3.73**	0.36	-0.90	0.19	-1.01
noise-clear AVia	-1.15	3.02**	2.69*	-2.92**	-0.40	-2.61*	0.92	-0.34
AVia	1.14	-3.02**	-2.69*	2.92**	0.40	2.61*	-0.92	0.34
AnVcAnVn	-2.17	3.99***	4.30***	-4.74***	-0.02	-2.48*	0.78	-0.96
AcVnAnVn	-1.02	3.07**	-2.70*	**-**0.48	-0.67	0.41	3.77**	-5.87***
AcVcAnVc	0.59	-1.19	-6.59***	3.65**	-0.11	4.11***	2.46*	-5.37***

Aud vs. base = auditory vs. baseline; Hi vs. fast freq. = high (1.87 kHz or 7.47 kHz) vs. low (0.12 kHz or 0.47 kHz) carrier frequencies; Slow vs. fast AM = slow (4 cycles/s) vs. fast (32 cycles/s) amplitude modulation; AV vs. fix = audiovisual stimuli vs. fixation cross; Clear vs. blurred = clear vs. blurred video clips; noise-clear AVia = audiovisual interaction in which the processing of noisy auditory information is enhanced with visual information; AVia = audiovisual interaction in which processing of blurred visual information is enhanced with auditory information; An = noisy auditory stimulus; Vn = noisy visual stimulus; Ac = clear auditory stimulus; Vc = clear visual stimulus. Statistically significant values are shown in bold. *p < 0.05, **p < 0.01, ***p < 0.001.

The occurrence rates of Patterns 2, 4, and 6 had the strongest positive correlations with the main effects indicating the processing of audiovisual information vs. baseline and auditory processing vs. baseline (z = 4.45–20.4, p < 0.001; Table 1). The occurrence rates of patterns 4, 6, and 7 correlated positively with the regressors, indicating the contrast between clear vs. noisy speech (z = 2.23–4.41, p < 0.05). Moreover, the occurrence rates of patterns 2 and 3 correlated positively with the regressors related to the processing of visual information and audiovisual interaction in which auditory information enhances the processing of blurred visual information (z = 2.61–4.30, p < 0.05).

## 4 Discussion

This study extends previous literature on auditory neuroscience that has mainly focused on static functional connectivity by showing that AC function can be characterized by a dynamically varying individual-specific set of coactivation patterns that share similarities during resting state and auditory stimulation. Our study also advances studies of dynamic functional connectivity by indicating that coactivation patterns can be identified within local AC networks, and they are, thus, not specific to brain-wide networks.

### 4.1 AC’s intrinsic activity can be characterized by a set of coactivation patterns

An increasing number of fMRI studies have identified dynamically reoccurring brain-wide coactivation patterns demonstrating that functional interactions between brain regions are not static over time but rather highly dynamic. The dynamically varying network configurations have been identified both during resting state and various dynamic tasks (Du et al., 2018; Gonzalez-Castillo & Bandettini, 2018). Thus, fluctuating patterns of interactions between distributed regions appear to be an intrinsic property of mammalian brain organization, which may facilitate the dynamic integration, coordination, and response to internal and external stimuli that are critical for ongoing cognition and behavior (Büchel & Friston, 2000; Tognoli & Kelso, 2009). Our results extend these previous studies of dynamic connectivity that have mainly focused on brain-wide networks by showing that dynamic coactivation patterns can also be reliably identified within local networks of AC from single time frames of the resting-state fMRI data. Furthermore, these results expand the auditory neuroscience research that has usually computed average functional connectivity across the whole scan, assuming connectivity to be static over time.

At the group level, the patterns were highly reproducible, as demonstrated by the average correlation of 0.96 ± 0.02 between the coactivation patterns and one between the cooccurrence rates of the patterns determined from two fMRI sessions of the same participants. The similarity was not explained by autocorrelation, as indicated by significantly lower similarities between the actual patterns from the first session and the permuted patterns from the second session. The individual-level reproducibility between the AC pattern occurrence rates was 0.86 which is lower than 0.90 reported previously for the whole brain (Peng et al., 2023). However, the signal-to-noise ratio and, therefore, also reproducibility may be expected to be lower for small AC networks than for large-scale networks. Furthermore, our results demonstrate that coactivation patterns can be identified within AC even without any a priori information provided by weighting with a group atlas, as was used in Peng et al. (2023).

There was still considerable similarity in the activation maps and their occurrence rates even when both the coactivation patterns and templates were determined independently within different datasets (Supplementary Fig. S5). Related results have been obtained in previous studies that have shown brain-wide coactivation patterns to share spatial similarities between datasets (Janes et al., 2020; Liu & Duyn, 2013; H. Yang et al., 2021). The AC coactivation patterns were also highly similar for the dataset with the templates derived from the same or fully independent dataset (Fig. 2).

While brain-wide coactivation patterns have usually been shown to be hemispherically symmetric (Huang et al., 2020; Janes et al., 2020; Li et al., 2024; H. Yang et al., 2021), the AC patterns showed notable hemispheric asymmetry. This likely reflects the lateralization of the AC functions (Tervaniemi & Hugdahl, 2003). In contrast, the brain-wide coactivation patterns are usually dominated by the default mode network, known to be hemispherically symmetric. However, with a large number of patterns, hemispheric lateralization has also been identified in brain-wide patterns. For example, a recent study (Peng et al., 2023) found one of the 16 patterns to be left-lateralized and one right-lateralized. The occurrence rate of the left-lateralized pattern was also higher during a language task than resting state and correlated with language-task onsets.

### 4.2 Coactivated patterns of AC are organized in pairs with opposite network topographies

The AC patterns formed pairs with opposite coactivation maps. There were no significant differences in the occurrence rates between the patterns and their opposite counterparts. If the pattern correlated positively with a task contrast regressor, its opposite pattern never correlated with the same task contrast or correlated with it negatively.

Opposite coactivation maps have previously been reported for brain-wide networks (Huang et al., 2020; Janes et al., 2020; Li et al., 2024; H. Yang et al., 2021). The opposite coactivation patterns may reflect anticorrelated networks commonly found in resting-state functional connectivity studies (Chai et al., 2012; Fox et al., 2005, 2009; Gopinath et al., 2015; Martinez-Gutierrez et al., 2022; Uddin et al., 2009). For example, the activity of the default mode network is decreased during the execution of cognitive tasks and increased during the resting state. Thus, the default mode network is also referred to as a task-negative network that often correlates negatively with task-positive networks (Fox et al., 2005; Liu et al., 2013). The strength of the negative correlation between the default mode network and the task-positive network has been associated with interindividual differences in task performance and task-evoked fMRI responses (Mennes et al., 2010). Our results extend these previous studies by demonstrating that anticorrelated networks are not specific to brain-wide networks, but can also be found within local networks of AC.

The meaning of the anticorrelated networks is unclear, but they may represent the separation of neuronal processes linked to competing cognitive demands, such as focusing on a task versus engaging in stimulus-independent thoughts (Fox et al., 2005). In this study, the anticorrelated AC networks may compete with each other while processing different features of auditory or audiovisual information. A rat study also found opposite patterns with opposite phases, suggesting that they may reflect peaks and troughs of periodic neural oscillations (Gutierrez-Barragan et al., 2019). While it has been proposed that these anticorrelations could be artifacts introduced by global signal (GS) regression (Murphy et al., 2009; Saad et al., 2012), growing evidence demonstrates the presence of anticorrelation patterns independently of preprocessing steps (Chai et al., 2012; Fox et al., 2005, 2009; Uddin et al., 2009). Our study further supports the neurophysiological origin of the anticorrelations since GS was not regressed out from the data.

### 4.3 AC coactivation patterns capture interindividual variability

Both the occurrence rates (interindividual similarity = 0.66) and spatial topographies (interindividual similarity = 0.58) of the coactivation patterns varied substantially between individuals. Within individuals, the occurrence rates (interindividual similarity = 0.86) and spatial topographies (interindividual similarity = 0.79) of the coactivation maps were more similar, indicating that the coactivation patterns are reproducible within individuals and the observed interindividual variability likely reflects true differences in the AC function rather than measurement noise. It should be noticed that the individual variability of the spatial topographies may also be affected by macroanatomical variability between participants because the normalization methods are less accurate in the areas that are not strongly related to major sulci and gyri (Frost & Goebel, 2013; Gulban et al., 2020). However, the between-participant similarity in the spatial topographies did not correlate with the between-participant similarity in macroanatomy within AC (curvature: r = −0.04, p = 0.62, thickness: r = −0.14, p = 0.88), suggesting that the individual variability in the coactivation maps is not explained by anatomical variability alone. Our results are in line with two recent studies that showed the brain-wide coactivation maps to vary substantially between individuals (Peng et al., 2023; H. Yang et al., 2023). Together with these results, our study expands previous studies that have mostly ignored individual variability of the dynamic coactivation patterns. Our results are also in accordance with recent static functional connectivity fMRI studies indicating that the functional organization of AC varies substantially between individuals (Hakonen et al., 2025; Luo et al., 2024) and that individual variability of the AC functional connectivity is even higher than in the visual cortex (Ren et al., 2021). Our results show that individual variability can not only be found in static functional connectivity of AC, but also in the occurrence rates and spatial topographies of the dynamically varying network configurations.

### 4.4 Processing of auditory information can be characterized with AC intrinsic coactivation patterns determined from resting-state fMRI data

The AC pattern coactivation maps shared similar network configurations when determined using group templates derived from resting-state and task data. We have previously demonstrated, using conventional GLM analysis on the same task data, that the stimuli elicited robust activation in the expected auditory regions (Hakonen et al., 2025). These results confirm that the task induced clear neural responses, reducing the likelihood that the similarity between resting-state and task-related patterns could be explained by weak stimulation. These results are in line with the studies that have found only small differences in the static functional connectivity networks between the resting state and task (Gratton et al., 2018; Krienen et al., 2014; S. M. Smith et al., 2009; Toro et al., 2008). Interestingly, we also found that the occurrence rates of AC coactivation patterns correlated with multiple task contrast regressors, suggesting that these patterns are involved in auditory and audiovisual information processing. Importantly, the occurrence rates were computed from the task data using group-level templates derived independently from resting-state data. The observed correspondence supports the view that task-evoked responses emerge from the brain’s intrinsic functional organization rather than being entirely stimulus-driven. One possible neurobiological explanation is that this organization minimizes the metabolic cost of transitioning into task-active states (Raichle, 2010). Our findings extend those of (S. M. Smith et al., 2009) by indicating that rest–task correspondence is an organizational principle of AC.

The topography of Pattern 2 displayed relative activation in the area comprising the posterior superior temporal gyrus (pSTG) and middle superior temporal sulcus (STS), and the activity was stronger in the left hemisphere (Supplementary Fig. S1). The posterior superior temporal cortex (pSTC) has been shown to be highly responsive to sound onsets, including onsets of speech and nonspeech sounds (Yi et al., 2019). In line with these results, Pattern 2 correlated most strongly with the regressors reflecting auditory stimulation vs. baseline and audiovisual stimulation vs. fixation cross. pSTG has also been shown to be important for audiovisual integration (Ozker et al., 2017; Ozker et al., 2018), which is again in line with our finding that Pattern 2 correlated with the regressor indicating an interaction where visual input enhances the processing of noisy speech and the regressor indicating clear vs. blurred visual information.

Patterns 4 and 6 were correlated with the regressors which reflect auditory information processing, but not with regressors related to visual or audiovisual processing. Patterns 4 and 6 were also correlated with the regressor, indicating Speech vs. noise contrast. The spatial topographies of patterns 4 and 6 showed relative activation in the most anterior parts of the planum polare and the primary auditory areas that are known to be highly responsive to acoustic information (e.g., frequencies, amplitude modulation, Hertrich et al., 2020; Lerner et al., 2011; Rauschecker & Scott, 2009). In the spatiotemporal topography of Pattern 4, the relative activation extended from the primary auditory areas to the anterior STS and temporal pole. These areas have been associated with the processing of speech, words, and semantics (Bonner & Price, 2013; Hertrich et al., 2020; Lerner et al., 2011; Rauschecker & Scott, 2009). In the spatiotemporal topography of Pattern 6, the activity was left lateralized for both Pattern 4 and Pattern 6.

The spatial topography of Pattern 7 showed left-hemispheric relative activity in the posterior STS. In contrast to states 3 and 5, the relative activity also expanded from the posterior STS to middle STG. In the right hemisphere, the relative activity was restricted in the middle STG for State 7 whereas for states 3 and 5 STG was deactivated compared to the other areas. Pattern 7 had the strongest correlations for regressors, indicating speech vs. noise and slow vs. fast AM, suggesting its role in speech processing. These results are in accord with previous literature showing pSTG, mSTG, and STS to be activated by words (Belin et al., 2002; Binder et al., 2000; Canolty et al., 2007; Dronkers et al., 2004; Fecteau et al., 2004).

The spatial topography of Pattern 3 displayed left-hemispheric relative activation in the pSTS. Pattern 3 was correlated with the regressors indicating clear vs. blurred visual processing and an audiovisual interaction where visual information enhances the processing of noisy speech. Pattern 3 did not correlate with any regressors specific to auditory or speech processing. Together, these results suggest that Pattern 3 could be important in the processing of audiovisual and visual information. This conclusion is consistent with studies that have associated left pSTS with the integration of audiovisual information (Beauchamp et al., 2010; Hickok et al., 2018; Kreifelts et al., 2007; Nath & Beauchamp, 2012). The pSTS has also been shown to be activated during lip-reading (Blank & von Kriegstein, 2013) and dynamic facial expressions (Venezia et al., 2017), which is further in line with our results as the audiovisual stimulus included video clips of a woman voicing “rain” or “rock”.

If the pattern correlated positively with the regressor, its opposite pattern correlated negatively with the same regressor or did not correlate significantly with it. These results are, again, in line with the studies that found anticorrelation within large-scale functional networks (Chai et al., 2012; Fox et al., 2005 2009; Gopinath et al., 2015; Martinez-Gutierrez et al., 2022; Uddin et al., 2009) and further support the conclusion that anticorrelated networks may also exist within local AC networks as discussed in Section 3.2.

### 4.5 Limitations

While this study provides valuable insights into the dynamically varying networks within the AC, it is important to recognize its limitations. First, no clearly optimal solution for the number of patterns emerged, as has often been the case also in studies analyzing whole-brain coactivation patterns (Huang et al., 2020; Karahanoğlu & Van De Ville, 2015; Liu & Duyn, 2013; Liu et al., 2013; H. Yang et al., 2021). In line with previous studies (Huang et al., 2020; Liu et al., 2013), different indices gave different recommendations for the number of patterns. However, the number of clusters did not have a strong impact on the coactivation patterns, as those identified with fewer clusters were also present in solutions with a larger number of clusters (Supplementary Fig. S3). Second, while in this pioneering study focusing on AC, we needed to follow previous procedures, a possible approach for further studies would be to collect more data from each participant (H. Yang et al., 2023). In this case, the individual variability could be taken into account by determining the template patterns individually which may increase individual-level reproducibility. Finally, the influence of inadequate registration of cortical surfaces on the individual variability of the AC patterns could be further decreased by using more sophisticated registration methods (Frost & Goebel, 2013; Gulban et al., 2020).

### 4.6 Conclusions and future directions

This study had four major findings: 1) AC function can be characterized by a set of dynamically varying coactivation patterns derived from single time-frames of the resting-state 7T fMRI data; 2) AC patterns derived from the resting-state and task fMRI data share a lot of similarities; 3) AC patterns get temporally synchronized with auditory stimulation, and 4) AC patterns capture interindividual variability. Identification of reproducible and relatively generalizable dynamic coactivation patterns advances previous research of AC functional organization that has mainly focused on static functional connectivity. By indicating that dynamically varying connectivity patterns are also highly individual, our results support and extend studies that have identified high individual variability in static functional connectivity of AC. Additionally, our results show that the dynamic AC network configurations remain relatively unchanged during the resting state compared to the task and get synchronized with specific features of auditory or audiovisual input. An important finding of our study is also that coactivation patterns are not specific to brain-wide networks but can also be found locally within AC. Interesting topics for further studies are associating the AC patterns with a more comprehensive set of auditory stimuli and investigating how individual variability in the AC patterns is reflected in auditory function, hearing abilities, and speech comprehension assessed with behavioral and clinical tests. An important question is also whether auditory processing problems could be identified from the AC coactivation patterns derived simply from the resting state instead of using several different auditory stimulus paradigms.

## Supplementary Material

Supplementary Material

## Data Availability

The data of the present study are available from the corresponding author upon request.
