# Supplementary material for: Function of the auditory cortex characterized by its intrinsic dynamic coactivation patterns estimated in individuals
Source: Imaging Neurosci (Camb). 2026 Mar 24;4:IMAG.a.1179. doi: 10.1162/IMAG.a.1179 (PMC13015430; doi:10.1162/IMAG.a.1179)
Supplement: Supplementary Material [file IMAG.a.1179_supp.pdf]

Supplementary material

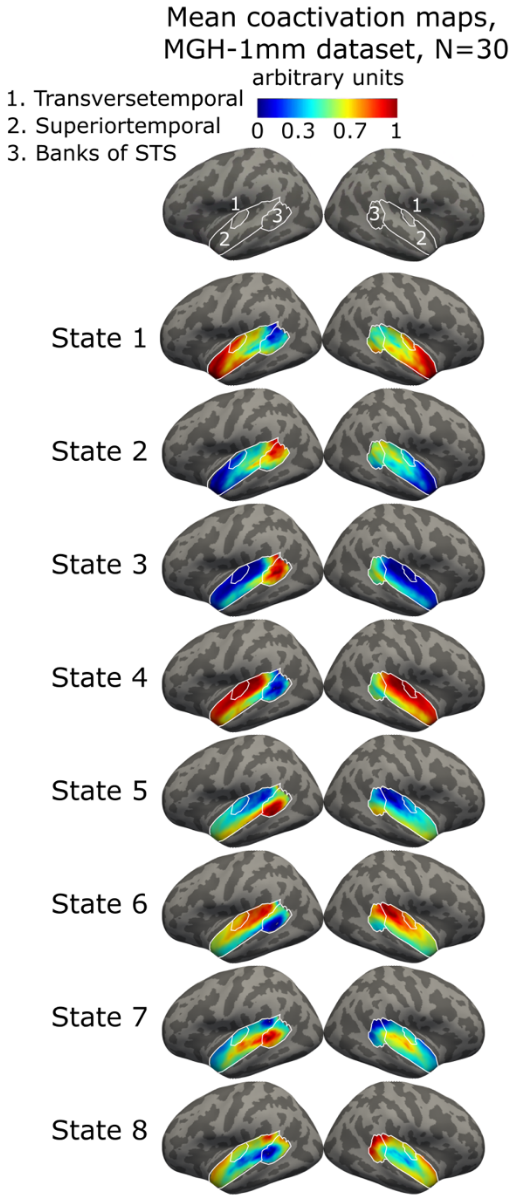

Figure S1. The correspondence between coactivation maps and anatomical organization of AC as defined by the Desikan atlas.

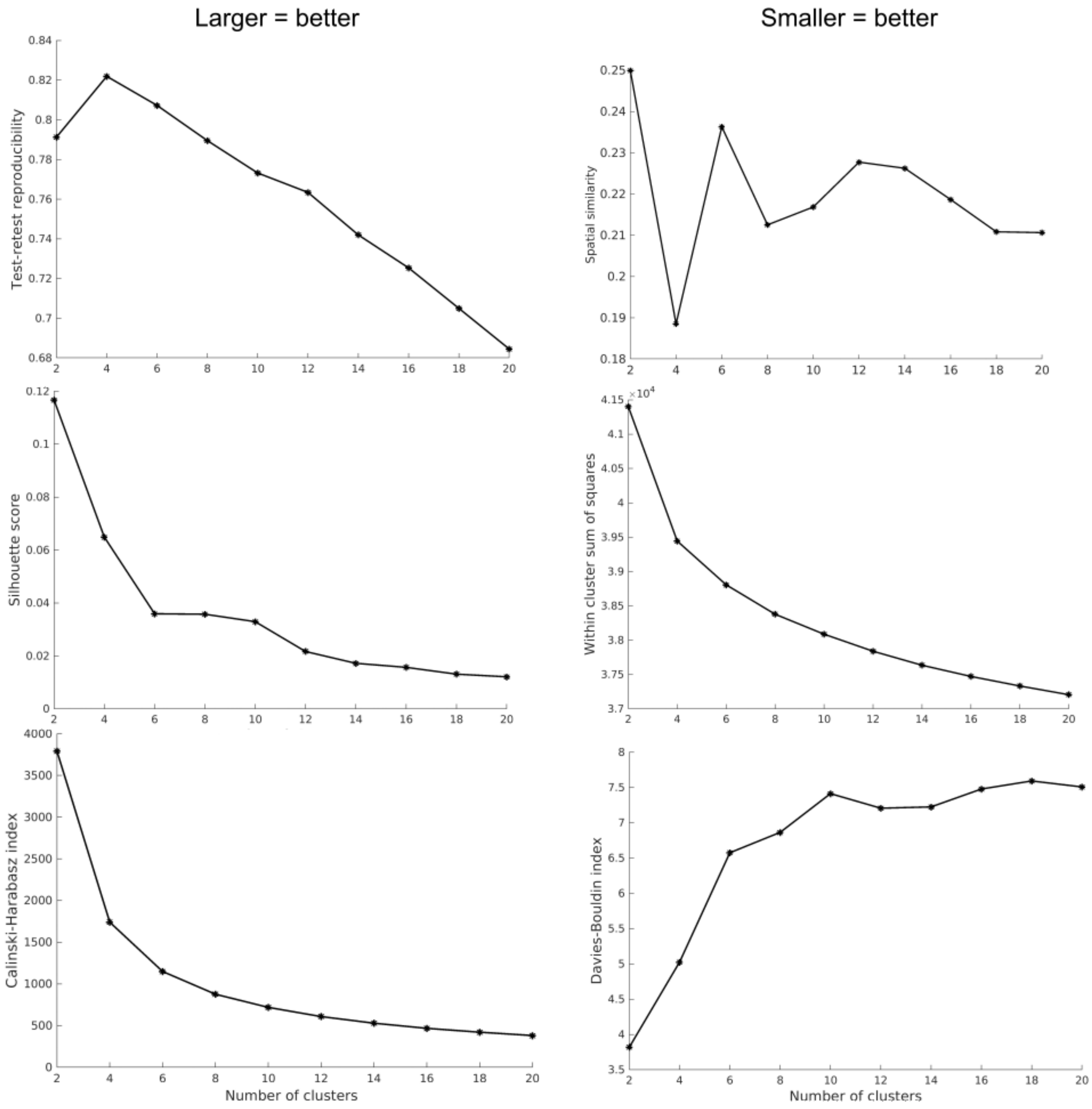

Figure S2. Estimating the optimal number of clusters from the resting-state data of the MGH-1mm dataset. For test-retest reproducibility, Silhouette score and Calinski-Harabasz index larger value indicates better clustering result. For Spatial similarity, Elbow method (i.e. within cluster sun of squares), and Davies-Bouldin index, smaller value indicates better clustering. Different methods produced inconsistent recommendations and none of them showed a single clearly best solution.

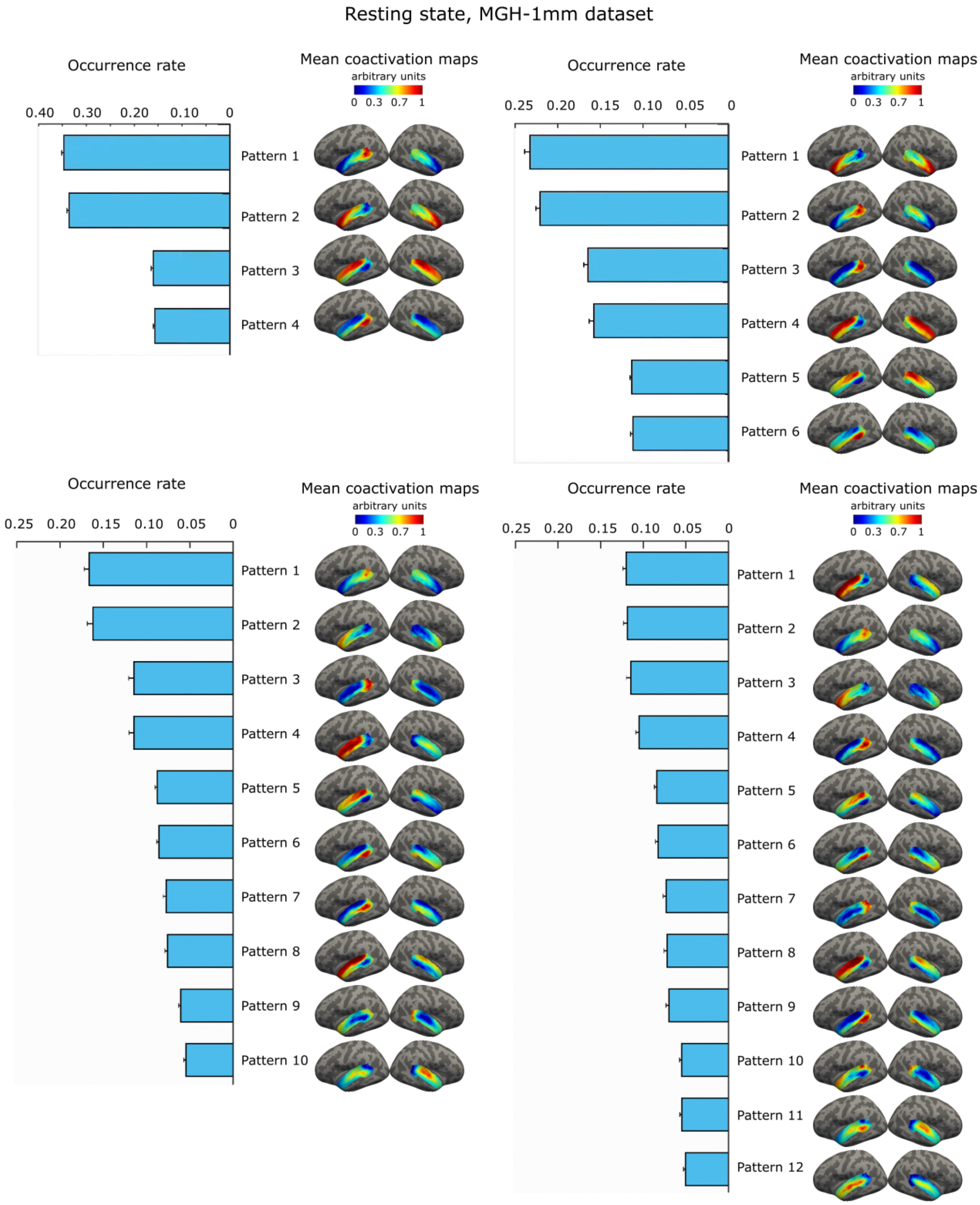

Figure S3: Occurrence rates and coactivation maps for 4, 6, 10, and 12 AC pattern solutions derived from the resting-state MGH-1mm data. The AC coactivation patterns were ranked by their co-occurrence rates in descending order. Error bars indicate the standard error of the mean (SEM).

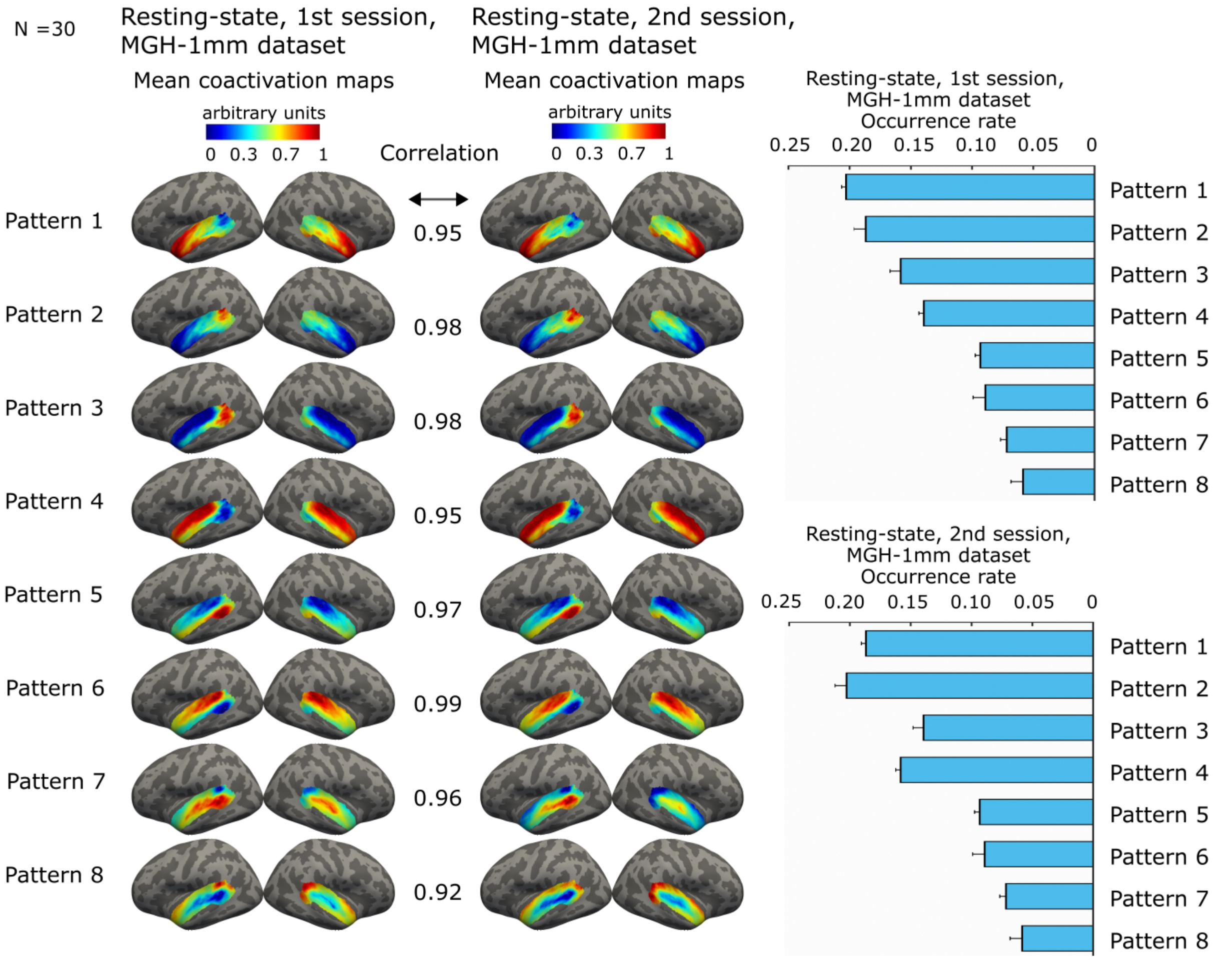

Figure S4: Reproducibility of the AC patterns between resting-state fMRI sessions of the MGH-1mm participants. Both the template coactivation patterns and the corresponding individual-level coactivation maps and occurrence rates were derived within the first and second resting-state sessions. The figure shows group-average results. The correlation values indicate the Spearman correlation between the corresponding patterns of the two datasets. All correlation values were statistically significant ($p<0.001$ for all patterns). The Pearson correlation between the occurrence rates was 0.96 ($p<0.001$). Error bars indicate the standard error of the mean (SEM).

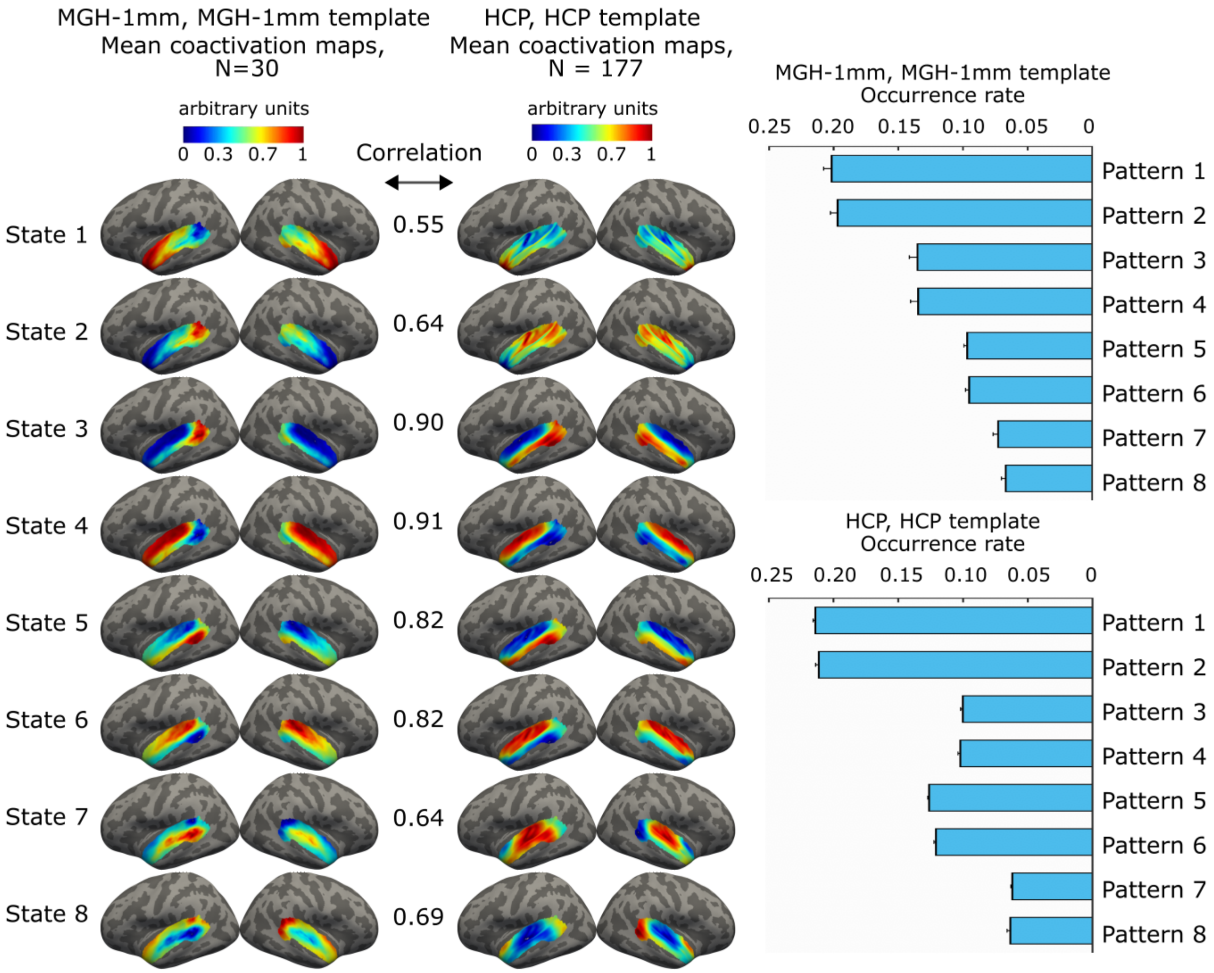

Figure S5: Generalizability of the AC pattern coactivation maps between datasets. The MGH-1mm maps were determined using the group templates from the same MGH-1mm data. The HCP maps were determined using the group templates from the same HCP data. The correlation values indicate the Spearman correlation between the corresponding patterns of the two datasets. All correlation values were statistically significant ($p<0.001$ for all patterns). The Pearson correlation between the occurrence rates was 0.91 ($p<0.002$). Error bars indicate the standard error of the mean (SEM).
